# Primary health care staff’s opinions about changing routines in practice: a cross-sectional study

**DOI:** 10.1186/1471-2296-15-2

**Published:** 2014-01-07

**Authors:** Siw Carlfjord, Karin Festin

**Affiliations:** 1 Department of Medical and Health Sciences, Division of Community Medicine, Linköping University, Linköping SE-581 83, Sweden

**Keywords:** Primary health care, Implementation, Staff opinions, Change

## Abstract

**Background:**

In health care organizations, there is a mutual interest from politicians, managers, practitioners and patients that the best available care is provided. Efforts are made to translate new knowledge and evidence-based practices into routine care, but there are a number of obstacles to this translation process. Factors related to the new practice as well as factors related to the implementation process are important, but there is still a knowledge gap regarding how to achieve effective implementation. The aim of the present study was to assess opinions about practice change among staff in primary health care (PHC), focusing on factors related to a new practice and factors related to the implementation process.

**Methods:**

A questionnaire was sent to 470 staff members at 22 PHC centres where a new tool for lifestyle intervention had recently been implemented. Thirteen items regarding the characteristics of the new practice and nine items regarding the implementation process were to be judged from not at all important to very important. A factor analysis was performed, and statistical analysis was done using the Kruskal-Wallis nonparametric test.

**Results:**

Four factors regarding the characteristics of the new practice were identified. Most important was *Objective characteristics*, followed by *Evidence base*, *Subjectively judged characteristics* and *Organizational level characteristics*. Two factors were identified regarding the implementation process: *Bottom-up strategies* were judged most important and *Top-down strategies* less important. The most important single items regarding characteristics were “easy to use” and “respects patient privacy”, and the most important implementation process item was “information about the new practice”. Nurses differed most from the other professionals, and judged the factors *Evidence base* and *Organizational level characteristics* more important than the others. Staff with more than 10 years experience in their profession judged the *Evidence base* factor more important than those who were less experienced.

**Conclusions:**

To incorporate new practices in PHC, objective characteristics of the new practice and the evidence base should be considered. Use of bottom-up strategies for the implementation process is important. Different opinions according to profession, gender and years in practice should be taken into account when planning the implementation.

## Background

In health care organizations there is a mutual interest from politicians, managers, practitioners and patients that the best available care is provided. Efforts are made to translate new knowledge and evidence-based practices into routine care, but there are a number of obstacles to this translation process. Such obstacles could be contextual factors, factors related to the intended adopters or characteristics of the new practice [1]. To overcome these obstacles, various strategies for implementation are discussed, and there is a growing interest in answering questions about what approaches should be used in which settings for which problems [2].

A number of theories and frameworks for implementation are described in the literature [1,3-5]. Damschroder et al. [6] presented a consolidated framework for implementation based on 19 published implementation theories. Five major domains are highlighted: intervention characteristics, outer setting, inner setting, characteristics of the individuals involved and the process of implementation. Intervention characteristics could be described as the characteristics of an innovation. Rogers [7] states that important innovation characteristics are relative advantage, compatibility, complexity, trialability, observability, and the possibility of re-invention. Re-invention, described as adaption to local circumstances, could be important to achieve a fit between the innovation and the adopting organization, which have also been shown to be important [1]. The evidence base of the new practice is another essential factor, stressed by Kitson et al. [8].

Strategies for implementation of evidence-based practices have been described by Nutley et al. [9]. They state that dissemination of research findings is important but not sufficient to change practice. More successful are strategies involving collaboration between researchers and practitioners, relying on influential experts or peers, or enabling the use of research through different kinds of support [9]. Incentives and reinforcements are other strategies mentioned, with mixed and limited evidence of success. The use of interventions tailored to overcome barriers to change in the receiving organization is advocated by Wensing et al. [10,11]. They suggest that different types of barriers to change can be addressed by using multicomponent interventions, and state that, in addition to science, some artistry is needed to choose or design an intervention. When tailored interventions for change in practice were evaluated by Baker et al. [12], they concluded that this strategy was more likely to improve professional practice than dissemination of guidelines or educational material. However, it is still unknown how to effectively identify barriers or how to select interventions likely to overcome these barriers [12].

In Sweden, primary health care (PHC) has an obligation to provide health promotion/preventive services, which requires a change from a disease-centred approach to a health promotion approach. A number of barriers to this change have been identified, such as lack of time, skills and resources [13,14]. To facilitate the delivery of health promotion in PHC, a computer-based lifestyle intervention tool (CLT), described in detail by Carlfjord et al. [15], was developed by a research team at Linköping University. A pilot study was conducted whereby the CLT was tried at a small number of PHC units, and feasibility, implementation strategies and staff experiences were evaluated [15-17]. To further explore factors that could influence the implementation of a new practice in PHC, and to enhance the possibility of tailoring implementation activities in the future, the research team saw a need to assess staff opinions regarding implementation in a larger sample. This was possible because after the small scale introduction, the CLT was offered to all PHC units in Östergötland County.

The aim of the present study was to assess opinions about practice change among staff members in PHC who recently have experienced the introduction of the CLT, focusing on factors related to a new practice and factors related to the implementation process.

## Methods

### Design and setting

The study was conducted among PHC staff in Östergötland County, Sweden, using a cross-sectional survey design. Östergötland County has about 420,000 inhabitants and has a mix of rural and urban communities. The county has been found to be representative of the Swedish population in terms of gender distribution, employment rates and economy [18]. Of the 42 PHC units operating in the county, nine participated in the pilot study [15]. The remaining 33 PHC units were invited to participate in the present study, and 22 agreed to participate. Unit size in terms of listed patients at the participating units varied from approximately 4000 to 18,000.

### Data collection

The CLT was introduced at the participating units in 2008-2010. After 2 years, a follow-up evaluation was performed using a survey questionnaire sent by e-mail to staff at the participating units. Staff groups included were general practitioners (GPs), nurses, assistant nurses (ANs) and allied professionals (APs). The questionnaire assessed opinions about using the CLT. It also contained two questions regarding general opinions about what is perceived to be more or less important when a new practice or tool is introduced in PHC. These questions focused on the characteristics of the innovation and factors related to the implementation process, and were based on former research described in the background to this article. The questions were put as “To make you incorporate a new practice, how important are the following characteristics of the new method?” (13 items), and “When a new practice is introduced at your work place, how important are the following factors?” (9 items). The questions were answered using a Likert type scale with the alternatives very important, important, less important, not important at all, for each of the suggested items. All the items from the two questions were included in the analysis.

### Data analysis

A factor analysis was performed to identify clusters of items, which could then be compared according to staff group, age and years of practice. The Kaiser-Meyer-Olkin measure of sampling adequacy was used to ensure that the sample size was adequate for these analyses. Direct oblimin rotation was used because the factors were expected to correlate. Factor loadings <0.4 were not reported. In addition, data from the questionnaires were analysed item by item, and answers from the different staff groups were compared using the Kruskall-Wallis or the Mann-Whitney *U* test. Statistical significance was set at *p* ≤ 0.05. However, when the four professional groups were compared, two to three analyses were conducted for each item, which might result in mass significance; therefore *p* values between 0.017 and 0.05 should be interpreted as tendencies rather than significant differences. Possible correlations between profession, years in practice and gender where tested with Spearman’s rho. Statistical analyses were performed using the computer-based analysis program SPSS version 21.0.

### Ethics

The study was carried out in compliance with the Helsinki Declaration and was approved by the Ethics Board in Linköping, Sweden (Dnr Ö 16-08). The participants were staff members therefore written consent was not required according to Swedish regulations (SFS 2003:460).

## Results

The questionnaire was sent to 470 staff members. Of those, 239 responded, yielding a 51% response rate. Internal drop-out for the questions used in the present paper resulted in response rates for the specific questions from 40% to 42%. An analysis of the drop-outs showed no differences between responders and the entire sample in terms of profession or gender, except that the proportion of GPs was lower among the responders (12.6%) than in the entire sample (20.5%).

### Characteristics of the new practice

The questions about importance of characteristics were analysed according to profession, years in practice and gender. The questions were answered by 27 GPs, 111 nurses, 19 APs and 37 ANs. Most were women (88%), and 83% had more than 10 years of practice in their current profession. Gender was associated with profession (*r* = 0.393, which is regarded as a moderate correlation), but associations between profession and years in practice, or gender and years in practice were very low.

The 13 characteristics focused on were as follows: How important is it that the new practice

– is evidence based?

– is in accordance with my personal values?

– has a relative advantage compared with current practice?

was tried and recommended by colleagues?

– is advocated in national guidelines?

– is advocated in local guidelines?

– is easy to learn?

– is easy to use?

– can be adapted to local circumstances?

– can be tried on a limited basis?

– is economically viable?

– is advocated by my immediate manager?

– respects patient privacy?

The Kaiser-Meyer-Olkin measure equalled 0.808, indicating that patterns of correlations were relatively compact so that a factor analysis should yield distinct and reliable factors [19]. Results from the factor analysis are presented in Table 1 and revealed four factors with an eigenvalue >1 regarding characteristics of the new practice (items included are mentioned in order of weight):

1. *Objective characteristics* (based on the items “easy to use”, “easy to learn”, “advocated in local guidelines” and “respects patient privacy”)

2. *Evidence base* (based on the items “advocated in national guidelines”, “advocated in local guidelines”, “evidence based”, “advocated by my immediate manager”)

3. *Subjectively judged characteristics* (based on the items “has a relative advantage…”, “in accordance with my personal values”, “tried and recommended by colleagues”, and “evidence based”)

4. *Organizational level characteristics* (based on the items “can be tried on a limited basis”, “is economically viable”, and “advocated by my immediate manager”)

**Table 1:** Results from the factor analysis of the questionnaire statements on the characteristics of the new practice

**Factor**	**Items**	**Loadings**	**Initial eigenvalue**	**Initial explained variance (%)**
Objective characteristics	Easy to use	0.96	4.50	34.7
	Easy to learn	0.90		
	Advocated in local guidelines	0.84		
	Respects patient privacy	0.55		
Evidence base	Advocated in national guidelines	0.85	1.66	12.8
	Advocated in local guidelines	0.79		
	Evidence based	0.58		
	Advocated by my immediate manager	0.44		
Subjectively judged characteristics	Has a relative advantage compared with current practice	0.80	1.33	10.2
	In accordance with my personal values	0.70		
	Tried and recommended by colleagues	0.54		
	Evidence based	0.42		
Organizational level characteristics	Can be adapted to local circumstances	0.74	1.03	7.9
	Is economically viable	0.61		
	Is advocated by my immediate manager	0.59		

When the four factors were analysed according to profession, age, gender and years in practice, it was found that nurses found the factors *Evidence base* and *Organizational level characteristics* more important than the other professionals (*p* = 0.002 and *p* = 0.001, respectively). No differences were found between GPs, APs and ANs. Women found *Organizationel level characteristics* more important than men did (*p* = 0.040). Staff with more than 10 years experience in their profession found the *Evidence base* factor more important than those who were less experienced (*p* = 0.013). Age was not associated with differences regarding the four factors.

**Figure 1:**
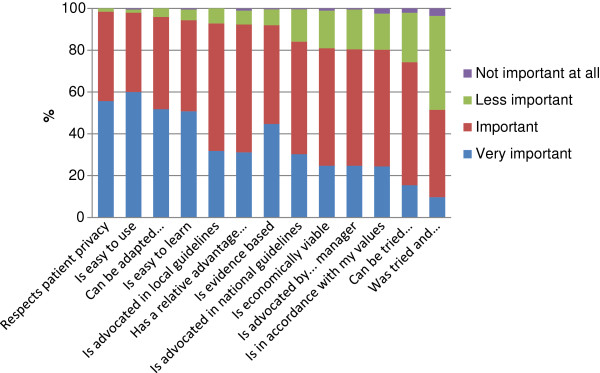
Proportion of responders who judged the importance of the characteristics according to the four levels displayed.

The 13 items representing characteristics of the new practice were also analysed one by one. Figure 1 shows how the responders judged each characteristic. In the figure, the characteristics are placed in order of perceived importance when the responses “very important” and “important” were combined, as a way to dichotomize the results, but still show how the answers were distributed among the alternatives. As shown in the figure, the characteristics considered most important were “respects patient privacy”, and “easy to use”. Less important was “was tried and recommended by colleagues”.

When groups were compared according to profession, gender and years in practice, significant differences or tendencies were found regarding five of the items, as shown in Table 2.

**Table 2:** Differences between groups regarding the characteristics of the new practice

**Characteristic of the new practice**	**Was more important to**	***p *** **value**
Is evidence based	Nurses than to ANs	0.001
Can be tried on a limited basis	Nurses than to GPs	0.007
	Nurses than to APs	0.018
	Women than to men	0.048
Is advocated by my immediate manager	Nurses than to GPs	0.001
	Nurses than to APs	0.009
	Nurses than to ANs	0.023
	More experienced* than to less experienced	0.014
Is advocated in local guidelines	Nurses than to ANs	0.048
	More experienced* than to less experienced	0.021
Is advocated in national guidelines	More experienced* than to less experienced	0.022

*More than 10 years experience in current profession.

### Implementation process

The question about importance of issues regarding the implementation process was answered by 189 individuals (27 GPs, 107 nurses, 19 APs and 36 ANs). Of these, 87% were women and 82% had more than 10 years of practice in their current profession. The nine issues were

– Influence over the decision to introduce the new practice

– Information about the new practice

– The manager being positive about the new practice

– Continuous support and encouragement from the manager (Manager support)

– Continuous support and encouragement from peers (Peer support)

– Continuous support and encouragement from a change agent outside the organization (Change agent support)

– Evaluation of the new practice after a certain amount of time

– Patients requesting the new practice

– Financial support to the PHC centre

The Kaiser-Meyer-Olkin measure was 0.756, which according to Hutcheson and Sofroniou [19] can be considered good. The factor analysis (Table 3) of the nine items above resulted in two factors with an eigenvalue >1 regarding characteristics of implementation practice (items included are mentioned in order of weight):

1. *Bottom-up strategies* (based on the items “the manager being positive”, “information…”, “continuous support and encouragement from the manager”, “influence over the decision…”, “continuous support and encouragement from peers” and “evaluation of the new practice…”)

2. *Top-down strategies* (based on the items “patients requesting…”, “continuous support and encouragement from a change agent outside…”, and “financial support”)

**Table 3:** Results from the factor analysis of the questionnaire statements on the implementation process

**Factor**	**Items**	**Loadings**	**Initial eigenvalue**	**Initial explained variance (%)**
Bottom-up strategies	The manager being positive about the new practice	0.84	3.78	42.0
	Information about the new practice	0.83		
	Continuous support and encouragement from the manager	0.81		
	Influence over the decision to introduce the new practice	0.74		
	Continuous support and encouragement from peers	0.69		
	Evaluation of the new practice after a certain amount of time	0.60		
Top-down strategies	Patients requesting the new practice	0.79	1.50	16.6
	Continuous support and encouragement from a change agent outside the organization	0.74		
	Financial support to the PHC centre	0.62		

When the two factors were analysed according to profession, age, gender and years in practice, it was found that the factor *Bottom-up strategies* was less important to GPs than to the other professions (*p* < 0.001). Furthermore, women scored higher than men did (*p* < 0.001). No differences were found according to age or years in practice. Regarding *Top-down strategies*, there were no differences according to profession, age, gender or years in practice.

**Figure 2:**
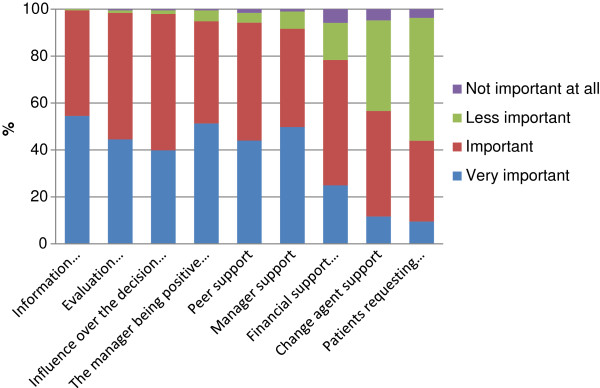
Proportion of responders who judged the importance of the issues according to the four levels displayed.

The nine issues were also analysed one by one. Figure 2 shows how the responders judged each issue. In the figure, the issues are placed in order of perceived importance. As shown in Figure 2, the item considered most important by the responders was “information about the new practice” and “patients requesting the new practice” was least important.

When groups according to profession, gender or years in practice were compared, significant differences or tendencies regarding eight of the nine items were found, as shown in Table 4. The new practice, “being requested by patients”, which was the item that was judged least important overall, showed most differences between groups.

**Table 4:** Differences between groups regarding implementation process issues

**Implementation process issue**	**Was more important to**	**p value**
Good information about the new practice	Nurses than to GPs	0.003
	Women than to men	<0.001
The manager being positive	Nurses than to GPs	<0.001
	ANs than to GPs	0.009
	Women than to men	<0.001
Support from the manager	Nurses than to GPs	<0.001
	ANs than to GPs	<0.001
	Women than to men	<0.001
Support from peers	Nurses than to GPs	0.001
	ANs than to GPs	0.019
	Women than to men	0.007
Support from a change agent outside the organization	ANs than to GPs	0.004
Evaluation of the new practice	Women than to men	0.010
The new practice being requested by patients	GPs than to nurses	0.022
	GPs than to APs	0.024
	Men than to women	0.037
	More experienced* than to less experienced	0.014
Financial support	Nurses than to GPs	0.005
	ANs than to GPs	0.029

*More than 10 years experience in current profession.

## Discussion

Staff opinions about factors important for changing practice, in relation to the characteristics of the new practice, and to the implementation process were assessed. The main finding was that *Objective characteristics of the new practice* were perceived as most important, and that *Bottom-up strategies* were given higher importance than *Top-down strategies*. Substantial differences between the professional groups were found regarding these factors, and some differences were found according to years in practice.

The two characteristics of a new practice that were considered most important regardless of staff category were that it should be easy to use, and that it should respect patient privacy. When Rogers [7] identified innovation characteristics important for diffusion, he mentioned complexity as a key attribute. The more complex the innovation, the lower rate of diffusion could be expected. From the present study, it is evident that complexity is not appreciated among staff and practices that make work easier are welcomed. The high value put on “Respects patient privacy” gives an indication of the prevailing patient-centeredness in health care [20]. Patients’ interests are considered very important, and a practice that threatens patient integrity would probably be rejected by staff. This issue would probably be more important among staff with higher education, but no such differences were found in the study. ANs, the group with lowest education level, did not differ from the other groups.

Differences regarding the characteristics of the new practice that were related to years in practice were whether the new practice was advocated in guidelines, and if it was supported by the immediate manager. Those who had long experience (10 years or more) were more likely to appreciate these features. When perceived competence to develop evidence-based practice was assessed in a Spanish study, it was found that nurses with shorter experience obtained the best scores [21], which could be seen as contradicting our results, and was explained by recent experience from university studies. A possible explanation for the findings in the present study could be that staff with long experience also have experienced new practices that have failed, and therefore seek methods that are recommended by some kind of authority. Results from the factor analysis stressed this issue even more, as the factor *Evidence base* was more important to staff with long experience in their current position than to their less experienced peers. The result, however, is surprising, taking into account the focus on evidence-based practice currently influencing education in medicine and nursing.

Another difference revealed from the factor analysis was that nurses find the factor *Organizational level characteristics* more important than the other professionals. This might reflect that nurses put themselves in a position of responsibility for the care delivered by the health care centre, they show loyalty to the manager and want things to work for everybody. Nurses are known to put a high value in the practice of nursing as described by Sellman [22], and a widened responsibility for nurses in terms of medical decision making is currently under discussion [23]. Nurses also differed from the other groups judging the factor *Evidence base* more important. Current nursing research is focusing extensively on evidence-based medicine or evidence-based practice, which could explain this difference.

A notable finding is that recommendation from peers was given low importance by the staff members in the present study. Influence from peers has previously been shown to be an important factor for organizational change in health care [24], but under the present circumstances, it seems to be less relevant. Qualitative research methods could be appropriate to explore this further.

With regard to issues most important for the implementation process, the two items “good information about the new practice” and “the manager being positive” were judged most important. Information is known to be important when changes are implemented in organizations [25], and the importance of the manager’s attitude towards change is also well documented in the literature [26]. Regarding these issues, the PHC staff members participating in the present study did not differ from staff in other organizations.

The issue given less importance when studying the entire group was “patients requiring the new practice”. However, GPs found it more important than the other groups, and those with long experience found it more important than the less experienced staff members. It could be that experienced GPs listen to a higher degree to patients’ preferences. This practice is in accordance with the original ideas of evidence-based medicine, stating that individual clinical expertise, the best available external clinical evidence from systematic research and patient preferences all are important to provide effective and efficient treatment [27].

Results from the factor analysis showed that the factor *Bottom-up strategies* was less important to GPs than to other professions. An explanation to this could be found in the hierarchical structure in health care, whereby GPs have a dominant position [28]. The dominant position of GPs might make them less interested in the bottom-up strategies as defined in this study; they seek information themselves and do not depend on the manager’s attitude or support.

In the present study, staff members in PHC were asked about their opinions regarding change in practice, and the answers could be influenced by social desirability. This should be taken into account when interpreting the results. However, as the study assesses opinions and not performance, the influence of social desirability is probably limited. The questionnaire used was developed exclusively for the present study, and face validity was obtained by discussing the questions among the members of the research team. The questions have not been tested for validity, which is a limitation. Another limitation of the study is that the response rates were not very high and GPs were underrepresented among the responders, which affect the generalizability of the results. A strength is that the study was performed shortly after the implementation of a new practice, which means that the responders all had recent experience of an implementation process.

## Conclusions

To incorporate a new practice in PHC, the objective characteristics of the new practice and the evidence base should be given consideration. It is important to use bottom-up strategies for the implementation process. Different opinions exist according to profession, but also according to gender and years in practice, which should be taken into account when an implementation activity is planned.

## Competing interests

The authors declare that they have no competing interests.

## Authors’ contributions

SC contributed to the design of the study, the acquisition and interpretation of the data, and was involved in drafting the manuscript. KF contributed to analysis of the data and was involved in drafting the manuscript. Both authors have read and approved the final manuscript.

## Authors’ information

SC has a PhD in Medical Science, holds a Master’s degree in Public Health and is a registered physiotherapist; KF has a PhD in Social Medicine and Public Health and holds a Master’s degree in Statistics.

## Pre-publication history

The pre-publication history for this paper can be accessed here:

http://www.biomedcentral.com/1471-2296/15/2/prepub
